# Implementation of evidence-based protocols improves survival: a 15-year surgical ICU experience in 10,172 patients

**DOI:** 10.1186/cc12402

**Published:** 2013-03-19

**Authors:** SA Nasraway

**Affiliations:** 1 Tufts Medical Center, Boston, MA, USA

## Introduction

There has been enormous interest in measuring ICU performance in terms of mortality and resource use, owing to increased public and health insurer scrutiny. We elected to describe the performance of our surgical ICU, using a standardized mortality ratio (SMR = observed / predicted mortality).

## Methods

The primary cohort was all patients admitted to the surgical ICU from March 2010 through February 2012 and related outcomes. The change in SMR was longitudinally determined from the latest 15-year study period (1997 to 2011) comprised of 10,172 patients.

## Results

**Figure 1:**
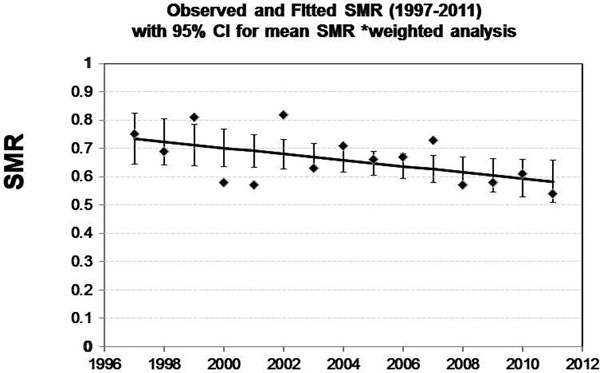
**Tufts surgical ICU, 15-year outcome standardized hospital mortality ratio**.

There were 1,799 ICU admissions in the primary cohort. Hospital mortality, observed and predicted by APACHE IV, was measured. Crude hospital mortality was 8.4%. The hospital SMR (observed / predicted mortality) was 0.58 (95% CI: 0.49 to 0.65). The SMR decreased by 20% from 0.73 to 0.58 over the 15-year study period, an absolute 1% per year decrease (*P *= 0.039; 95% CI: -0.02 to -0.002), as shown in Figure 1.

## Conclusion

Mortality was less than predicted and steadily declined during the previous 15 years. The SMR can be used to track success when new quality measures are introduced or changes in the delivery of care are made. ICUs should report their standardized mortality ratios to evaluate performance.

